# Doll play improves false belief reasoning: Evidence from a randomized-control trial

**DOI:** 10.1371/journal.pone.0343698

**Published:** 2026-03-18

**Authors:** Sarah A. Gerson, Jennifer Keating, Salim Hashmi, Ross E. Vanderwert

**Affiliations:** 1 Cardiff University Centre for Human Developmental Science (CUCHDS), School of Psychology, Cardiff University, Cardiff, United Kingdom; 2 School of Social Sciences, Cardiff University, Cardiff, United Kingdom; 3 Department of Psychology, Institute of Psychiatry, Psychology and Neuroscience, King’s College London, London, United Kingdom; 4 Cardiff University Brain Research Imaging Centre (CUBRIC), School of Psychology, Cardiff University, Cardiff, United Kingdom; Father Muller Charitable Institutions, INDIA

## Abstract

Play is often described as a child’s “occupation,” both because young children spend the majority of their time playing when given the option and because it is a critical mechanism through which children learn both cognitive and socio-emotional skills. In a randomized control trial (N = 73), we found the first causal evidence that doll play, more so than creative tablet play, improves false belief understanding in 4- to 8-year-old children following a six week long play intervention. Improvements in false belief were particularly strong for children who had more parent-reported peer problems. Consistent with prior research, children were more likely to play socially with dolls than socially with tablets during the intervention period and were more likely to use internal state language about others when playing with dolls than with tablets (when observed in the laboratory). Together, this shows that doll play may be an important play pattern for practicing and improving social processing skills like false belief reasoning. The mechanism underlying this improvement requires further investigation, but we speculate that dolls may encourage social interaction and further practice of these skills outside of social interactions.

## Introduction

Theory of mind (ToM), or the ability to understand and differentiate the internal states of both oneself and others (i.e., thoughts, beliefs, and desires), is a foundational skill for interacting with, learning from, and developing relationships with peers, teachers, and parents, with eventual implications for successfully engaging with colleagues, romantic partners, and business partners [1–6]. Research suggests that acquiring a rich understanding of others’ minds is a complex and prolonged endeavor, with some going so far as to suggest that the capacity to understand others’ minds and the propensity to use this skill in social interactions is uniquely human [7–10]. One of the reasons that the development of these skills in human children is thought to be an enduring process is because of the rich, complex, and bidirectional nature of factors associated with theory of mind skills, including language, executive function, and social experiences [11,12]. In the current research, we focus specifically on the role of social experiences and interactions via doll play in the development of false belief reasoning.

Research examining social influences on the development of theory of mind have focused on interactions with caregivers [13–16], siblings [17–19], or peers [20–23]. Within these social interactions, some have recognized the unique role that play may afford for learning about and practicing social cognitive skills [19,24–26]. Play is an important part of children’s development as it is a critical mechanism through which they learn both cognitive and socio-emotional skills [27]. In particular, pretend play has been proposed to be related to theory of mind acquisition due to their reliance on similar skills: 1) the ability to think of one object as being two things at once, 2) the ability to think of one object as representing another, and 3) the ability to have mental representations [28]. Play offers opportunities to learn both through the enactment of pretend play itself and through interactions with play partners. Perner et al. [29] found indirect evidence that social interactions during play might promote ToM. They found that children with one or two siblings were twice as likely to pass a false belief task than only-children; leading them to speculate that siblings may promote false belief understanding via acting as play partners and increasing engagement in pretend play. Indeed, Perner and colleagues [1] suggest that “pretend play is perhaps our best candidate for a cooperative activity which furthers the eventual understanding of false belief” (p. 1236). A broad literature suggests that children from larger families (e.g., more siblings) tend to have better theory of mind skills due to the presented opportunities for varied social interactions in these contexts [11,30,31]. Taken together, these findings highlight that playing with others might be one mechanism through which children’s ToM develops.

Associations between pretend play and theory of mind have also been found *without* social play partners. For example, children who have imaginary companions have been found to demonstrate better emotion understanding and theory of mind than those who do not have an imaginary companion [32,33]. The authors suggest that this may be the result of imaginary companions providing children with an opportunity to practice their ToM skills [32], however, it may also be the case that children with more sophisticated ToM more readily create and play with imaginary companions raising questions about the causal direction of this association. Further, the presence of an imaginary companion is relatively rare (a meta-analysis reported 22.71% of children had imaginary friends [34]]). A more widespread phenomenon that allows play with imagined others occurs when children play with toys like dolls. Dolls are often considered “social toys” [35] in that children tend to choose to play with others when given these kinds of toys [36,37]. The affordances of these kinds of toys also allow children to create fantasy scenarios even when playing alone with dolls that similarly offer them the opportunity to think about other’s beliefs and perspectives [38]. In this way, pretend play with dolls has the potential to encourage an understanding of others.

Previous research has provided preliminary support for the notion that doll play may be a promising avenue for promoting social processing skills like theory of mind. A recent neuroimaging study measured activity in an area of the brain associated with social processing, theory of mind reasoning, and social engagement (posterior superior temporal sulcus, pSTS) [39,40] while children spontaneously played with dolls and tablets both alone and with a social partner (an experimenter). This research found similar pSTS activity when children played with a partner regardless of the types of toys with which they played [41,42]. This was not particularly surprising given that the pSTS is active when both adults and children engage in social interactions [40,43], but it was the first evidence that this brain region is activated when children engage in semi-naturalistic interactions with others. More importantly for the current research, this same region was also active when children played *alone* with dolls, more so than when they played alone with tablets [41,42] highlighting the social nature of dolls. In a follow-up study, Hashmi et al. [44] found that children used more internal state language (ISL) – verbalizations that refer to the thoughts, feelings, and desires of others – when playing with a partner *and* when playing alone with dolls more so than playing alone with tablets, thus mirroring the pSTS findings. Individual differences in the use of ISL about others was also associated with pSTS activation [44]. These findings suggest that doll play may simulate aspects of social interactions that improve theory of mind otherwise present in play with siblings, peers, and parents, even when children are playing alone.

One of the reasons the development of ToM abilities are central to early development is because they have important bidirectional associations with children’s concurrent and subsequent social interactions. Even in toddlerhood, negative peer interactions at 24 months were associated with less ToM understanding at 36 months [23]. The opposite is also true: more sophisticated ToM during childhood influences how children interact with their peers [45]. A meta-analysis by Slaughter and colleagues [45] found that children with higher ToM scores were more popular amongst peers. Similarly, Abdullah et al. [1] observed that increased ToM scores were associated with a decrease in parent-reported peer problems. ToM abilities provide children with the tools to communicate and resolve conflicts with their peers more effectively, with long-term consequences for peer relationships and associated aspects of mental health and wellbeing [46]. In addition, there is also evidence that children with more traits reflecting social communication challenges often associated with peer problems (e.g., autistic traits) seek out a play partner when playing with dolls, but not tablets, alone [47]. Taken together, we predict that children with more peer problems at baseline may benefit more from playing with dolls than their peers with fewer problems because imaginary play and imagined interactions provide them with a predictable environment to practice social information processing, for example, as indicated through the use of ISL.

Previous studies [41,44] identified an association between engaging in doll play and social processing skills with regards to language and brain activity. It is, as yet, unknown whether doll play actually improves social processing abilities like theory of mind and false belief. Thus, the current study aims to examine whether there is a causal effect of doll play on false belief. We used a randomized-control trial design to allow us to identify whether there was a causal effect of doll play, controlling for individual factors, natural maturation, and other, less social creative play (tablets), on false belief reasoning improvement. We intentionally used a naturalistic design to estimate the potential causal effects of various kinds of play. That is, we sent families home with one of two types of toys (dolls/creative tablet games) and encouraged them to play with them at least three times a week, but we allowed them freedom to choose how to play with these toys and did not further restrict their play outside of this. We asked parents to report on their child’s play activity (frequency and social context) using online play diaries and transcribed their language during play sessions in the laboratory before and after the intervention to provide a clearer picture of how children played with each type of toy. We expected to replicate prior research indicating that children use more ISL about others during doll play than tablet play [44]. Further, we hypothesized that children assigned to play with dolls during the home intervention would show greater improvement on the false belief task during their return visit relative to children who were assigned to play with tablets at home. Given the bidirectional links between ToM and peer interaction discussed above, we might expect that children with greater social difficulties (e.g., more peer problems) may benefit more from engaging in doll play during the intervention.

## Materials and methods

### Participants

Eighty-one children aged 4–8-years-old were recruited via social media and a database of local families (South Wales, UK) interested in research to take part in this study (Mean age = 6.09 years, SD = 1.34, 42 Females). One child did not complete their first session, leaving a sample of 80 children. Parents confirmed that their child did not have any diagnosis of a neurodevelopmental condition or any developmental delays. Parents who did not want their children playing with either dolls or tablets were excluded. Data collection began on the 02/08/2022 and was completed 31/08/2023.

Upon enrolling in the study, children were randomly assigned to one of two conditions: doll or tablet. At the first visit, children assigned to the Doll condition chose three Barbie/Ken dolls of their choice (along with some accessories) to play with at home; children in the Tablet condition were given a tablet with three pre-loaded games (see Procedure section for more details) to play with at home. Of the 80 children who completed the first session, 42 were in the Doll condition, and 38 were in the Tablet condition. The groups did not differ in terms of age (p = .729) or sex (p = .832). 73 children completed their Time 2 visit (Mean age = 6.26 years, SD = 1.33, 39 Females). Of these, 38 were in the Doll condition and 35 were in the Tablet condition (see Table 1 for demographic information). Parents completed written consent forms and children provided verbal assent at the start of both sessions. The study was reviewed and approved by the University Ethics Committee.

**Table 1:** Demographic information of children who provided data at both time points.

	Whole sample (*n* = 73)	Doll (*n* = 38)	Tablet (*n* = 35)	*p*
Age at T2 (years)	6.26 (1.33)	6.26 (1.35)	6.25 (1.32)	.95
Sex				.42
Male	34	16	18	
Female	39	22	17	
Ethnicity				.57
White	66	35	31	
Asian/Asian British	1	0	1	
Mixed Race	6	3	3	
Annual household income	£58,271 (£28,870)	£57,500 (£31,652)	£59,188 (£25,650)	.81
Highest level of education				.79
≤ Secondary School	9	5	4	
≥ Bachelor’s Degree	63	32	31	

Values in parentheses are the standard deviations. *p* = *p*-value when calculating difference between Doll and Tablet groups. Not all parents completed information about their education level.

### Measures

#### Parent questionnaires.

**Play Diaries.** Parents were sent a short play diary to fill out online every 3 days during the play at home period of the study. This questionnaire consisted of 6 questions, asking parents how often their child played with dolls and tablets (information on both dolls and tablets were collected regardless of the condition the child was assigned to) over the past three days. Parents reported if their child had played with dolls/tablets for: less than 30 minutes, 30–60 minutes, 60–90 minutes, 90–120 minutes, or more than 120 minutes. Parents also reported who their child played with (alone, friend, sibling, parent, other; they could check multiple social contexts if their child played with more than one kind of social partner) and whether they used the materials provided to them or played with their own toys (e.g., using their own tablet/doll sets). In order to assess whether or not children effectively engaged with their assigned toy, we added the minimum number of minutes the child played with the assigned toy across the reported observations (e.g., 0 for less than 30, 30 for 30–60 minutes, 60 for 60–90 minutes, etc.) as a conservative measure of amount of play. We also created a ratio score to use as a measure of differential engagement with the assigned toy relative to the non-assigned toy: Time spent playing with assigned toy-type [i.e., dolls in Doll condition and tablets in Tablet condition]/time spent playing with non-assigned toy-type. A copy of the play diary used is included in Supplementary Materials (https://tinyurl.com/PeerReviewSupp).

**Strengths and Difficulties Questionnaire (SDQ)** [48]**.** Parents completed the SDQ, a parent-report measure of child mental health for 4–17-year-olds. Parents rate their endorsement for each item on a Likert scale from 0 (not true) to 2 (certainly true). The SDQ consists of 5 subscales, each with 5 items: hyperactivity problems, conduct problems, peer problems, emotional problems, and prosocial behaviors. Higher scores indicate more difficulties, except for the prosocial behaviors scale which is inversed. In the current study, only the peer problems and prosocial scales are analyzed, given our interest in links between social processing and peer interactions.

#### Behavioral assessments.

**Laboratory Play Sessions.** During pre- and post-intervention laboratory visits, children engaged in play with both male and female dolls with diverse races, size, and clothing/accessories. They also played on tablets with games that included Hoopa City 2 (Dr. Panda, Chengdu, China), in which players build roads, buildings, lakes, etc. to create a city for animated characters and Toca Hair Salon 3 (Toca Boca, Stockholm, Sweden), in which children can choose characters and wash, cut, and style their hair. These tablet games were chosen as they offer open-ended game play, without strict rules or objectives, and include characters similar to the open-ended possibilities of doll play [1].

Children’s use of ISL was coded from the transcripts of children’s speech using a coding scheme developed by Paine et al. [49] and used in previous studies with similar experimental procedures [42,44]. This scheme captures references to seven different categories of internal states (*cognition, desire, emotion, intention, preference, perception,* and *physiology*) and the referent of the internal state (*self, character,* or *other*). Each four minute play session was coded in 5-second segments, and multiple categories and referents could be coded within each of those segments. For the present analyses, children’s ISL was collapsed across internal state categories, and we combined children’s references to the internal states of ‘characters’ and any ‘other’ individual. The frequency counts were summed across the two solo doll play and two solo tablet play conditions. An independent observer, blind to the child’s intervention group assignment, coded the frequency of children’s use of ISL for a random subsample of 28 of the transcripts of children’s play across pre- and post-intervention sessions and conditions (approximately 20% of sessions). Intraclass Correlation Coefficients for ISL about characters and others ranged from.93 to.99.

**Sandbox Task.** The Sandbox Task used in the current study was adapted from Bernstein et al. [50] and used to measure false belief. Unlike conventional false belief tasks, this task assesses a continuous measure of ToM, resulting in variable performance across the full age range in the current study. In the task, an experimenter introduces two characters, one of whom buries an object in a sandbox (first location). This character then leaves and the other moves the object to a new location (second location). The participant is asked to mark where the first character will look for the object (i.e., a false belief question) and the distance between their mark and the correct answer (first location) is calculated. This yields a continuous measure of how much children are influenced by their own knowledge of the object’s location versus the character’s false belief. The task also includes control memory trials, which ask the child where the object was originally hidden without accounting for beliefs. These memory trials account for errors that are unrelated to false belief reasoning and are due instead to the linguistic and working memory challenges presented by the task. This control is particularly important given the variability of executive functioning in this age range [51].

**Fig 1:**
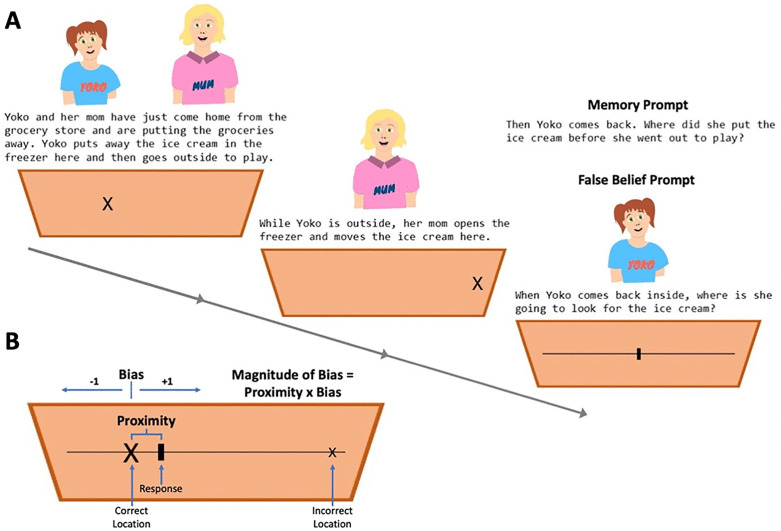
Schematic of the Sandbox task. **(A)** Flow diagram of the progression of each story with the letter X indicating the location the object was first placed and where it was moved. Locations were counterbalanced in terms of side of screen and ranged from 140 and 440 pixels away from one another. Note that similar setups and stories were used for false belief and memory trials, with the only difference between these being whether the prompt asked about the character’s mental states or physical actions. **(B)** Example trial response and how proximity, bias, and magnitude of bias scores were computed.

In the current study, the story was presented on a screen, with each location marked as an X in a 2-D picture of a Sandbox (Fig 1A). Children completed four trials – two memory and two false belief trials using a mouse to indicate their answer, with experimenter assistance dragging the mouse when necessary. The order of trials was counterbalanced across participants. The stories and characters were adapted from Sommerville [52]. Children were shown one set of stories during Time 1 and a separate set of stories during Time 2. A *proximity score* was calculated as the difference in pixels between the child’s response and the correct (first) location. To account the direction of their response and whether they demonstrated an egocentric bias, we also calculated a *magnitude of bias* score for each trial. Bias was coded as positive if their response was in the direction of the second location relative to the first location or negative if the response was in the opposite direction of the second location (i.e., no egocentric bias). The magnitude of bias was computed as the product of the bias and proximity score (Fig 1B). These scores were then averaged across the two trials for both false belief and memory trials, creating an average magnitude of bias score for each type of trial, with higher numbers indicating more egocentric bias (i.e., higher scores on false belief trials are indicative of poorer false belief understanding).

### Procedure

During their first visit to the lab, children engaged in a series of 4-minute play sessions with two different kinds of toy types (dolls and tablets) and across two social contexts (joint and solo). They always began with two four-minute joint play session in which an experimenter played with them. Whether they played jointly with a tablet or dolls was counterbalanced between participants. After the two joint play sessions, they engaged in four solo play sessions, alternating between doll play and tablet play (order counterbalanced between participants; see [47]. All sessions were video recorded for later transcription and ISL coding.

Children completed the Sandbox task after the 35-minute free play task. The stories for the Sandbox task were presented on an iiyama ProLite 2400 LCD monitor using E-Prime 3.0 (Psychology Software Tools, PA, USA) and children sat at a round table in front of the monitor. Before each trial, the child watched a 10-s baseline of five pseudorandom images of clipart fruits presented on a black background in the center of the screen for 1.5 s each, interspersed with a white fixation cross displayed for.5 s. This was part of an fNIRS recording procedure for a larger study but unrelated to the current results. An experimenter narrated each story for the child, beginning with an introduction to the characters, introducing a location change, and then asking them either the memory or false belief prompt, depending on the trial type. A second experimenter changed the screen to the next part of the story through a button press once the previous part had been read to the child. The child answered each question by dragging the small black rectangle along the Sandbox to their desired location with a computer mouse.

Upon completion of the first session, each child was randomly assigned to take home a tablet with three preloaded games on it (see description above in materials; note: same games played during laboratory session as well as Toca Kitchen 2 (Toca Boca, Stockholm, Sweden), in which players can create their own recipes and dishes) or to choose 3 Barbie and Ken dolls with accessories (Mattel Co., El Segundo, CA, USA, more information in supplementary materials; https://tinyurl.com/PeerReviewSupp). Parents were instructed to encourage their children to play with their assigned toy at least three times a week (with no upper limit) for an intervention period of approximately five to seven weeks. The design of the intervention was to be as simple as possible, relying on the ways children naturally play with the toys rather than prescriptive activities. Therefore, we did not constrain whether the child should play alone or with others and parents were not instructed to encourage any particular type of play with the study stimuli, limit any other types of play, or change any other aspect of their child’s play activities. We asked parents to report play activities (i.e., duration and social context) in their play diaries. The hypotheses of the study were not shared with parents, although they were aware that their child would be randomly assigned to one of two conditions.

On their second visit (approximately 5–8 weeks later), children took part in the play session and Sandbox task using the same procedure described for Time 1. Upon completing their second visit, children in the doll condition were offered a small prize worth approximately £5 and children in the tablet condition were offered a small prize and a doll to take home.

### Statistical approach

We utilized an intent-to-treat (ITT) approach to our analyses. ITT means that every participant is analyzed as a member of their randomly assigned group and assumes full participation in the intervention (whether or not this is true). This approach has multiple advantages. ITT ensures that the internal validity of the RCT design (i.e., eliminating sample biases and equal distribution of potential confounding factors within each group; [53,54] remains intact. Additionally, any effects of the intervention that are observed reflect what would be expected in a real-world implementation of the intervention [53,55,56].

## Results

### Intervention engagement

We first examined the fidelity of intervention implementation within and across intervention groups. The duration of the intervention was 7.20 weeks on average (SD = 3.01), with no significant difference in duration between the Doll and Tablet intervention groups (*p* = .144). Parents submitted an average of 8.88 (SD = 3.34) play diary entries over the intervention period (difference between groups not significant; *p* = .608). Parent report indicated that children played with their assigned toy (doll in the Doll condition and tablet in the Tablet condition) for at least 230 minutes across the intervention on average (at least 37 minutes per week on average), implying that the intervention was effectively implemented. Additionally, a one-sample t-test comparing the intervention ratio score to 1 (i.e., equal time playing with assigned and non-assigned toy-type), revealed that children spent more time playing with the assigned toy-type than the unassigned toy-type within each condition (Doll: *p* = .018, Cohen’s *d* = .426; Tablet: *p* = .006, Cohen’s *d* = .516). Children tended to use the study materials more than their own doll and tablet toys across both conditions (Doll: p = .019, Cohen’s *d* = .416; Tablet: *p* < .001, Cohen’s *d* = 1.070). Across groups, parents reported that children were more likely to play alone with tablets than with dolls (*p* = .009, Cohen’s *d* = .327) and more likely to play with sibling(s), parent(s), or friend(s) with dolls than with tablets (*p*s < .03, Cohen’s *d*’s ≥ .27).

### Improvement in false belief reasoning

**Fig 2:**
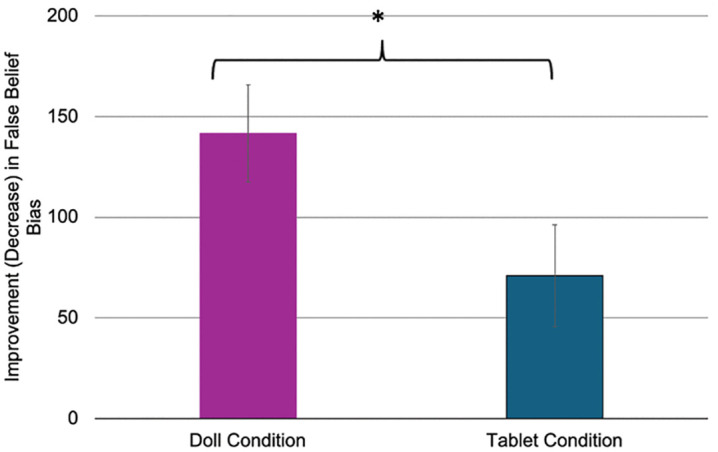
Improvement in bias (in pixels) between Time 1 and Time 2 for both Doll and Tablet intervention groups. This difference was significant for improvement in false belief (*p < .05) but was not significant for improvement in memory.

In order to assess the extent to which children’s performance on the sandbox task changed from pre- to post-intervention, we calculated a difference in magnitude of bias score (Change in Bias Score) separately for both false belief trials and memory trials by subtracting the magnitude of bias at Time 2 from the magnitude of bias at Time 1. Given that higher scores at each time point indicate more bias (i.e., more influenced by the child’s knowledge about the actual location of the object than by the protagonist’s false belief about the actual location), a positive difference score indicates a decrease in bias from Time 1 (pre-intervention) to Time 2 (post-intervention). Although we had no reason to believe age would have an effect on the outcome of the intervention, we included age as a covariate given that false belief varies greatly within this wide age range [57]. Relatedly, we also added sex as a between-subjects factor to control for any possible effects of sex on the intervention and/or false belief performance [58,59]. The False Belief Change in Bias Score was entered into a Univariate Analysis of Variance as the dependent measure, with intervention group (2: Doll, Tablet) and sex (2: male, female) as between-subjects factors and age (at Time 2) as a covariate. This revealed a significant effect of group (*F*(1,68) = 5.20, *p* = .026, η*p*^2^ = .071; Fig 2), a marginal effect of age (*F*(1,68) = 2.89, *p* = .094, η*p*^2^ = .041), and no other main effects or interactions (*p*s > .17).

In order to confirm that the effects were specific to false belief rather than general improvement on the task or in executive functioning, we ran the above analysis with Memory Change in Bias Score (to account for working memory challenges independent of false belief) as the dependent measure instead of False Belief Change in Bias Score. No significant effects emerged in this model (*p*s > .27). Additionally, when Memory Change in Bias Score was added as a covariate to the above model with False Belief Change in Bias Score as the outcome, no effect of Memory Change in Bias Score was detected (*p* = .20) and the effect of group remained (*p* = .040, h_*p*_^2^ = .062), suggesting that the effect was specific to false belief.

### Individual differences in false belief reasoning improvement

**Fig 3:**
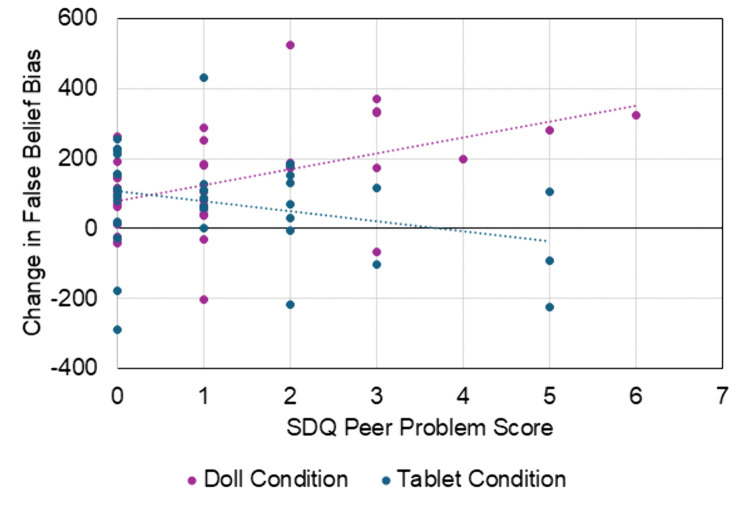
Relation between improvement in false belief from Time 1 to Time 2 (higher scores indicate more improvement) and peer problem scores for each condition.

To investigate whether individual differences in terms of peer relationship problems or prosocial behavior related to improvement in false belief reasoning, we conducted separate, exploratory correlations within each condition (Doll vs Tablet intervention to maintain ITT) between False Belief Change in Bias Score (note: higher scores indicate more improvement from time 1 to time 2) and peer problems and prosocial as parent-reported in the SDQ. Due to skew in the data (one-sample Kolmogorov-Smirnov for peer problem scores, *p*s < .001), Spearman’s correlations are reported. In the Doll intervention condition, a positive correlation between False Belief Change in Bias Score and peer problems was revealed (*r*_s_(37) =.474, *p* = .003; Fig 3). For the Tablet intervention condition, no relation was found (*r*_s_(35) = −.253, *p* = .14). These correlations were significantly different from one another (Z = 3.05, *p* = .002). No significant relations between False Belief Change in Bias Score and prosocial behavior was found in either condition (*r*_s_ < .10, *p*s > .59).

Further exploratory correlations investigated whether any parent report of play behavior was related to improvement in false belief within either condition. No significant correlations between false belief improvement and frequency of play with assigned toy or social context of play were revealed for either condition (*p*s > .18; see supplementary materials for full correlation matrix: https://tinyurl.com/PeerReviewSupp).

### Play sessions in lab

**Fig 4:**
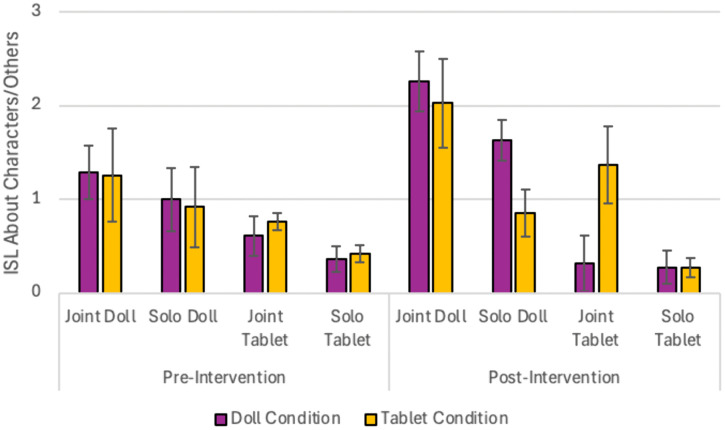
Differences in Internal State Language (ISL) about others use across different play conditions for each intervention group during the pre- and post-intervention laboratory play sessions.

To assess whether we replicated previous research regarding children’s use of ISL during various kinds of play (Hashmi et al., 2022), we explored children’s ISL about others (or characters) during the sessions in the laboratory at both pre- and post-intervention. At pre-test, a Repeated Measures 2 X 2 ANOVA with play type (doll/tablet) and social context (joint/solo) as repeated measures and intervention group (Doll or Tablet) as a between-subjects factor revealed a main effect of play type, *F*(1,70) = 10.07, *p* = .002, η*p*^2^ = .13, a marginal effect of social context *F*(1.70) = 3.3, *p* = .074, η*p*^2^ = .045, and no other main effects or interactions (*p*s > .59). Across both intervention groups, there was more ISL about others during doll play (*M* = 1.09, *SEM =* .20) than tablet play (*M* = .53, *SEM* = .12) and marginally more ISL about others during joint play (*M* = .98, *SEM* = .18) than solo play (*M =* .64, *SEM* *= .*14; Fig 4). When sex and age were added as between subject and covariate variables, there were no additional interactions or effects (*p*s > .19).

The same analysis at post-test revealed a similar main effect of play type, *F*(1,71) = 22.94, *p* < .001, η*p*^2^ = .24, and social context, *F*(1,71) = 11.93, *p* < .001, η*p*^2^ = .14. Additionally, a significant interaction between play type and intervention group emerged, *F*(1,71) = 4.71, *p* = .033, η*p*^2^ = .062, as well as a marginal interaction between social context and intervention group, *F*(1,71) = 3.52, *p* = .064, η*p*^2^ = .047. No other main effects or interactions emerged (*p*s > .49). Follow-up pairwise comparisons revealed that the play type x intervention group interaction was driven by a larger difference in ISL about others between doll and tablet play sessions for the Doll intervention group (mean difference = 1.65, *SEM* = .33, *p* < .001) than for the Tablet intervention group (mean difference = .62, *SEM* = .34, *p* = .074). In contrast, the Doll intervention group showed no difference in ISL about others between joint and solo contexts (mean difference = .34, *SEM* = .30, *p* = .36), but this difference was significant for the Tablet intervention group (mean difference = 1.14, *SEM* = .31, *p* < .001), with children in the Tablet intervention group using more ISL about others during joint play than solo play. When sex and age were added as between subject and covariate variables, there were no additional interactions or effects (*p*s > .22).

## Discussion

This study used a randomized-control trial to explore whether playing with dolls over an extended period of time improved theory of mind reasoning in a false belief paradigm. We found evidence that children assigned to play with dolls improved more on a false belief task than children assigned to play with tablets during the home intervention period. This effect was consistent for both boys and girls and across ages over a developmental period when theory of mind is being refined. Random assignment of children to the two different intervention groups ensures equal distribution of other factors that may relate to false belief reasoning, including cognitive ability, language ability, and other developmental phenomenon developing during this age period. As the only factor that consistently varied between groups was the type of toy each child was given and encouraged to play with at home, we can be reasonably confident that the unique improvement in false belief reasoning is due to the type of assigned toy, suggesting causal evidence that doll play improves false belief reasoning. Interestingly, given the bidirectional relations between peer relations and social cognition found in the literature [60–62], we also found greater improvements in false belief reasoning across time points in children with more parent-reported peer problems.

This study is the first to examine whether doll play has direct benefits for the development of social cognition, thus providing an important foundation for recognizing the value of this kind of play and raising important questions about the scope of these benefits and the mechanisms underpinning them. Although it is beyond the scope of the current study to pinpoint the causal mechanisms, we propose two speculative mechanisms based on prior research and exploratory data from this study: [1] doll play may encourage children to engage in social interactions more than other kinds of play [35–37,47], and [2] children use more ISL during doll play, offering an opportunity to rehearse or reflect on other’s beliefs, emotions, or intentions [44,63].

Doll play may encourage social interactions more than other kinds of play. In previous research investigating doll and tablet play in a neurodiverse sample [47], children higher in autistic traits tended to try to initiate talk with an adult in the room when asked to play alone with dolls more so than when asked to play alone with tablets. In the current research, we embraced the spontaneous variability in social interactions prompted by different kinds of play and found results consistent with this pattern. That is, reports from parent diaries of play at home during the intervention period found that children were more likely to play with dolls with a social partner (sibling, parent, friend, or other) and more likely to play with tablets by themselves. This was true regardless of intervention condition, gender, or reported peer problems, suggesting that it may reflect a broader play tendency in everyday life. This is supported by previous research that has found that children play more socially with dolls and other pretend play items (e.g., dollhouses and puppets) than with puzzles, books, and other play items [35–37]. Additionally, other research has found that children are less likely to engage socially when playing on digital devices than tangible devices [64,65]. Based on this, dolls might encourage more solitary children to engage in social interactions that encourage them to think and talk about others’ mental states. This natural variability may be one mechanism through which dolls improve false belief reasoning.

Another potential mechanism for doll play improving false belief reasoning is that children are more likely to use ISL when playing with dolls, relative to when playing with tablets, and when playing with others, relative to when playing alone [44]. In the current research, we replicated this finding when children engaged in play within the laboratory session before and after the home intervention. Specifically, before the intervention, both groups of children consistently used more ISL about others when playing with dolls than when playing with tablets. They also showed a trend to use more ISL when playing jointly with an experimenter than when playing alone. Following the intervention, these same effects of play type (more ISL during doll play than tablet play) and social context (more ISL during joint than solo play) were replicated, but we also identified an interaction between play type and intervention group such that children in the Doll intervention group showed a greater difference between ISL about others between doll and tablet play than the Tablet intervention group. Thus, the intervention also affected the way children in the Doll intervention group spontaneously played with dolls; elaborating on a key play pattern intrinsic to dolls. Taken together, it is plausible that doll play offers opportunities to practice thinking and talking about others’ mental states.

While we speculate on the possible mechanisms through which doll play can support social cognition, dolls have other affordances. For example, when playing with dolls, children have the opportunity to role play characters, create narratives, and act out scenarios [38], all of which rely on, but may also foster, the ability to imagine other’s thoughts, feelings, and intentions [28]. Through these pretend play scenarios, children may practice social skills, emotion processing, and emotion regulation, within a safe, controlled environment (see [66] for similarities about reading literary fiction). Previous research confirms this notion in that children both engage social processing brain regions and use more ISL about others when playing alone with dolls than with tablets [41,44,47]. In this research, solo doll play mimicked social interactions (i.e., when children played with a social partner with either dolls or tablets) in terms of both linguistic and neural activity associated with mentalizing. Further, this opportunity for rehearsed social interactions without another person present may be particularly helpful for children with peer problems, as children with peer problems may find peer interactions challenging or unpleasant. Future research will be necessary to fully characterize the various features of doll play to gain a complete understanding of the specific mechanisms underlying the sociocognitive benefits of doll play and whether these extend to other toys that may similarly prompt the creation of narratives around characters and mental states.

One strength of the present randomized control trial design is that we used an active control case rather than a passive control, so we had the chance to capture the unique benefits of certain kinds of play (i.e., play with humanoid dolls) relative to other play (i.e., creative open-ended play with tablets) [67]. This, however, leaves open the possibility that rather than doll play promoting social interactions and improving false belief reasoning, tablet play could be inhibiting social interactions and ToM. While this is possible, there are several reasons we think this is unlikely based on both the current data and prior research findings. First, in the present study, individual differences related to parent-reported peer problems only had an influence on false belief reasoning improvements in the Doll condition and not the tablet condition. No individual differences in terms of frequency of play or the social nature of play were related to individual differences in false belief improvement in the tablet intervention group. Additionally, we believe that social play in and of itself is not the critical active ingredient to practicing social interaction skills, rather, that combined with the act of mentalizing and use of internal state language during play is the underlying mechanism. Previous research has shown references to the internal states of others and associated neural activity was not different during social play with the tablet and dolls [44]. Therefore, it is unlikely that playing with tablets with others inhibits the opportunity to reflect on the internal states of others. In fact, children in the Tablet intervention group used ISL about others during joint tablet play at post-intervention (see Fig 4). The tablet games used for this study were chosen based on their creative and open-ended play and the integration of characters into the games, to match those aspects of doll play (i.e., no set task or goal). Of course, there are still many dimensions of play that differ between tablets and dolls, including two versus three dimensional engagement and the fine motor skills required for play. Although the chosen tablet games did not seem to encourage social interaction as much as dolls, other tablet activities (e.g., multiplayer “party games” or online interactions) may encourage social play, and in return could show similar social development benefits to doll play. Relatedly, future research should consider whether other kinds of creative play more closely align with dolls.

The implementation of our randomized-control trial reflected a natural intervention that resulted in a conservative estimate of the effects of doll play on false belief reasoning. Although we ensured that participants were given opportunities to play with a particular set of toys and encouraged to do so, we also embraced the natural variability in children’s play and did not prevent children from playing with non-intervention toy-types. Because of this, not every child played with their assigned toy more than the unassigned toy, yet we did find a group effect of more play with the assigned than unassigned toy overall. Importantly, our ITT analytic approach ensured we avoided introducing biases or other confounds into the data [53,54,56] and randomization was effective in producing evenly matched groups in terms of age, sex, ethnicity, household income, and parent education. Taken together, finding a group effect on false belief despite these factors and possible variability in play type suggests that an intervention with greater adherence to doll play would likely produce improvements in children’s false belief reasoning. Moreover, that we found an effect using a procedure that embraced the natural variability of children’s play and the availability of other toys holds promise for real-world interventions.

We asked parents to complete diaries to report secondary data on their child’s play during the intervention period. We saw variability in terms of adherence and most children would have also played in settings outside the home not captured by the parent, so we cannot be certain about the extent to which this secondary diary data is reflective of the true play patterns of the children. Additionally, the play diaries did not include audio or video recordings that allowed coding of the quality or type of play engaged in by the children. There was thus likely a great deal of variability in how children played at home and the extent to which they used internal state language [44]. We attempted to overcome this limitation by measuring ISL during different kinds of play in the laboratory as a proxy for what they may have done during the home intervention period. In doing so, we replicated previous patterns of ISL use, but future research should verify this is a true reflection of the play occurring in a non-laboratory setting.

These findings raise new and important questions regarding the scalability of this intervention across school and community settings. A recent pretend-play training program improved 5–6-year-olds’ emotion comprehension and found a decrease in aggressive behavioral responses [68]. However, Richard and colleagues [68] study required 20 hours of teacher training. Simply giving children dolls to play with would be a more cost-effective strategy for improving false belief reasoning, but before investing in this kind of intervention, replication and extension would be fruitful. In the current research, we did not collect data on aggressive behavior but only collected data on changes to false belief at one time point immediately post-intervention. Further, while our sample was representative of the community in which the research was conducted, it is difficult to assess whether the effect of doll play would generalize to broader communities, span other social behaviors, or be maintained over a longer developmental period is currently unknown.

The current findings are novel in identifying the first evidence of a causal effect of doll play on false belief reasoning. Prior research [49] has found associations between false-belief understanding and other measures of children’s broader theory of mind abilities [11]. Given the importance of social cognition for long-term outcomes spanning social, academic, and emotional domains [1–6], identifying new and innovative ways to improve skills like false belief reasoning could have important consequences. As noted in the introduction, by no means do we suggest that doll play is the only avenue to improving false belief reasoning. Instead, theory of mind development is a highly complex and prolonged skillset, with dynamic factors both contributing to and being influenced by its development [11,12].
